# Evaluation of the Microbial Diversity in Amyotrophic Lateral Sclerosis Using High-Throughput Sequencing

**DOI:** 10.3389/fmicb.2016.01479

**Published:** 2016-09-20

**Authors:** Xin Fang, Xin Wang, Shaoguo Yang, Fanjing Meng, Xiaolei Wang, Hua Wei, Tingtao Chen

**Affiliations:** ^1^Department of Neurology, The First Affiliated Hospital of Nanchang UniversityNanchang, China; ^2^Institute of Translational Medicine, Nanchang UniversityNanchang, China; ^3^State Key Laboratory of Food Science and Technology, Nanchang UniversityNanchang, China

**Keywords:** high-throughput sequencing, amyotrophic lateral sclerosis (ALS), microbial diversity, principal coordinate analysis (PCoA), central nervous system (CNS)

## Abstract

More and more evidences indicate that diseases of the central nervous system have been seriously affected by fecal microbes. However, little work is done to explore interaction between amyotrophic lateral sclerosis (ALS) and fecal microbes. In the present study, high-throughput sequencing method was used to compare the intestinal microbial diversity of healthy people and ALS patients. The principal coordinate analysis, Venn and unweighted pair-group method using arithmetic averages (UPGMA) showed an obvious microbial changes between healthy people (group H) and ALS patients (group A), and the average ratios of *Bacteroides*, *Faecalibacterium*, *Anaerostipes*, *Prevotella*, *Escherichia*, and *Lachnospira* at genus level between ALS patients and healthy people were 0.78, 2.18, 3.41, 0.35, 0.79, and 13.07. Furthermore, the decreased Firmicutes/Bacteroidetes ratio at phylum level using LEfSE (LDA > 4.0), together with the significant increased genus *Dorea* (harmful microorganisms) and significant reduced genus *Oscillibacter*, *Anaerostipes*, *Lachnospiraceae* (beneficial microorganisms) in ALS patients, indicated that the imbalance in intestinal microflora constitution had a strong association with the pathogenesis of ALS.

## Introduction

Amyotrophic lateral sclerosis (ALS) belongs to idiopathic, fatal neurodegenerative disease of the human motor system (Gordon, 2011), characterized by the loss of neurons at all levels of the motor system—from the cortex to the anterior horn of the spinal cord (Kiernan et al., 2011). The scientific and clinical interest in ALS is growing since the 1990s, and survival in ALS is now understood to be dependent on clinical presentation (phenotype), rate of disease progression, early presence of respiratory failure, and the nutritional status of patients (Kiernan et al., 2011; Fang, 2015). Unfortunately, less than 50% of patients can survive within 3 years of onset (Gordon, 2011; Kiernan et al., 2011; Fang, 2015).

The human gastrointestinal tract is home to bacterial communities, and the microbes have profound implications on human metabolism, immunity and the gut-brain axis (Derrien and van Hylckama Vlieg, 2015; Sivan et al., 2015; Yu et al., 2015; Zhernakova et al., 2016), and numerous studies have highlighted interactions between the central nervous system (CNS) and the gastrointestinal system (Erny et al., 2015; Wang et al., 2016). The brain may modulate peripheral gut functions to modify the gastrointestinal composition via releasing gut factors (hormones, neurotransmitters, immune factors), and the gut microbes, on the other hand, interact with the CNS by releasing of neurotransmitters, e.g., nitric oxide (NO, a major neurotransmitter in the brain) and g-aminobutyric acid (GABA, neurotransmitter produced *Lactobacillus* and *Bifidobacterium*) (Rhee et al., 2009; Barrett, 2014; Williams et al., 2014; Cani and Knauf, 2016). Short-chain fatty acids (SCFAs), the specific metabolites generated by gut bacteria, can cross the blood–brain barrier and its levels in the feces could be correlated negatively or positively (Serre et al., 2015) with autism spectrum disorders (ASD) (Adams et al., 2011). Moreover, researchers found that lipopolysaccharide (LPS), a constituent of Gram-negative bacteria markedly affected vagal afferent neuron function, with reduced vagal afferent leptin signaling (Serre et al., 2015).

Intestinal barrier dysfunction may promote the passage of toxins in the intestinal lumen into the blood, and the innate immune response and increased circulating LPS play pivotal roles in the pathogenesis of ALS (Nguyen et al., 2004; Zhang et al., 2009). Furthermore, reduced tight junction proteins in the lumbar spinal cord, as well as the disruption of tissue barriers (the blood–spinal cord barrier and the blood–brain barrier) were identified both in ALS patients and animal models (Longstreth et al., 2005). However, the interaction of the gut microbiota with the ALS has not been investigated.

In the past, only a small fraction of all bacteria have been isolated and characterized severely limited by available technology and the shortage of reference genomes (Yue-Xin et al., 2003; Shokralla et al., 2012), and recent technological advances in next generation sequencing technology has enabled elucidation of the pleiotropic effects of microorganisms on the human host (Derrien and van Hylckama Vlieg, 2015; Sivan et al., 2015; Yu et al., 2015; Zhernakova et al., 2016). In the present study, the high-throughput sequencing analyses were used to assess the interaction of the gut microbiota and the ALS, which proves basic data for the prevention and treatment of ALS.

## Materials and Methods

### Ethical Statement and Patients

The study was approved by the Ethical Committee of The First Affiliated Hospital of Nanchang University, all participants provided written informed consent and all the methods were carried out in accordance with the approved guidelines.

Six consecutive patients with ALS (according to the revised El Escorial criteria) were recruited (Brooks et al., 2000) at The First Affiliated Hospital of Nanchang University between 07/2015 and 05/2016, and patients who were unable to communicate, either verbally or by writing, were excluded. None had additional neurological disease or previous mental illness. Respiratory function measured by forced vital capacity (FVC) was above 70% and there was no evidence of nocturnal hypoventilation (Supplementary Table  S1). Five healthy people without ALS were recruited as control. All people with random diets donated their first fecal motion of the day for only one time and the samples were stored at -70°C.

### Extraction of Genomic DNA and High-Throughput Sequencing

Genomic DNA from each sample was extracted using a TIANamp Genomic DNA kit (TIANGEN) combined with bead beating as previously published (Yu et al., 2015). Then the Genomic DNA was sent to the one of the most famous high-throughput sequencing company for high-throughput sequencing and analysis.

The extracted genomic DNA was used as the template to amplify the V3–V4 region of 16S rRNA genes using the primer pair 338F/806R with the barcode. PCR reactions, pyrosequencing of the PCR amplicons and quality control of raw data were performed as described previously with minor modification (Xu et al., 2015).

### Bioinformatics and Multivariate Statistics

Low-quality sequences were eliminated from analysis based on the following criteria: (a) raw reads shorter than 400 bp; (b) a sequence producing more than eight homopolymers; (c) >2 mismatches in the primers, or, (d) one or more mismatches in the barcode. Pyrosequenced amplicons were removed using the PyroNoise algorithm in Mothur (Schloss et al., 2009). Bioinformatic analysis was implemented using the Quantitative Insights Into Microbial Ecology (QIIME) platform (Caporaso et al., 2010). Briefly, 16S rRNA operational taxonomic units (OTUs) were clustered using an open-reference OTU picking protocol based on 97% nucleotide similarity with the UCLUST algorithm (Davenport et al., 2014). ChimeraSlayer was employed to remove chimeric sequences (Haas et al., 2011). The relative abundance of each OTU was determined as a proportion of the sum of sequences for each sample. Taxonomic relative abundance profiles (such as, at the phylum, class, order, family, and genus levels) were generated based on OTU annotation. The microbial community structure (i.e., species richness, evenness and between-sample diversity) of bacterial samples was estimated by biodiversity. Shannon index, phylogenetic diversity, Chao1 index, and the observed number of species were used to evaluate alpha diversity, and the weighted and unweighted UniFrac distances were used to evaluate beta diversity.

All of these indices (alpha and beta diversity) were calculated by the QIIME pipeline.

### Statistical Analysis

Statistical analysis was implemented using the R platform. Principal coordinate analysis (PCoA) was performed using the “ape” package based on the UniFrac distances between samples. The difference among groups was further assessed using a non-parametric test via Metastats software^1^ as described previously (Lu et al., 2014), and statistical significance was set at *p* < 0.05 for correction of multiple comparisons.

## Results

### Sequencing Coverage

To compare the fecal microbes of healthy people (group H) and ALS patients (group A), 16S rRNA amplicon sequencing analysis was used to sequence the V3–V4 hypervariable region, and the sequencing data was filtered to get the valid data, and all the effective tags of all samples were clustered and those sequences with over 97% similarity were considered as one OTU. In total, 802695.96 filtered clean tags (72972.36 tags/sample) and 2540 OTUs were obtained from all the samples with an average of 230.91 OTUs per group (**Table 1**). Chao1 index had almost got saturated and the rarefaction curve of every sample could enter the plateau phase (Supplementary Figure  S1).

**Table 1:** Number of raw tags, clean tags, average bp, OTUs, and actual bacterial composition in groups A and H by high-throughput sequencing.

Sample ID	Raw Tags	Clean Tags	AvgLen (bp)	OTU
A1	97751	82942	452	291
A2	147122	132071	452	269
A3	104463	88679	457	301
A4	109556	94243	457	206
A5	92412	80310	457	145
A6	100046	85055	461	190
H1	48699	36373	446	239
H2	46394	36601	449	231
H3	72834	53787	448	218
H4	84893	61487	447	224
H5	80500	51148	457	226
Average	89515.45	72972.36	453	230.91

### Shared Genera in Each Sample

**FIGURE 1:**
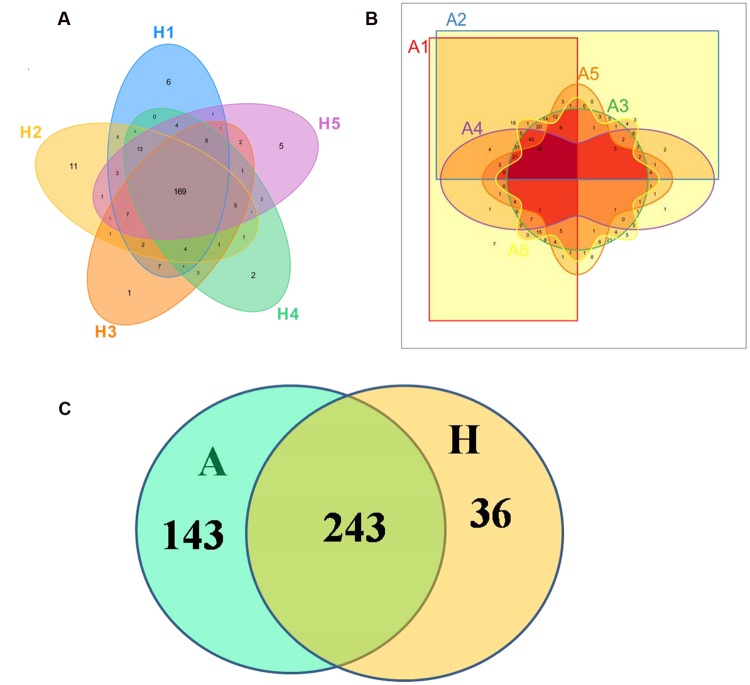
**Scalar–Venn representation of the microbiota between groups A and H. (A)** Shared OUTs among samples H1, H2, H3, H4, and H5. **(B)** Shared OUTs among samples A1, A2, A3, A4, A5, and A6. **(C)** Shared OUTs between groups A and H.

The Venn figure could reflect the difference between group A and group H. As shown in **Figure 1**, there were 386 and 279 OTUs in group A and H, and the percent of their common OUTs were 63.0% (243/386) and 87.1% (243/279), respectively. For group H, 43.78% OTUs (169/386) were identified as common OUTs among samples H1, H2, H3, H4, and H5, while the common OTUs only occupied 17.56% (49/279) among samples A1, A2, A3, A4, A5, and A6.

### The β Diversity of the Microbial Community

**FIGURE 2:**
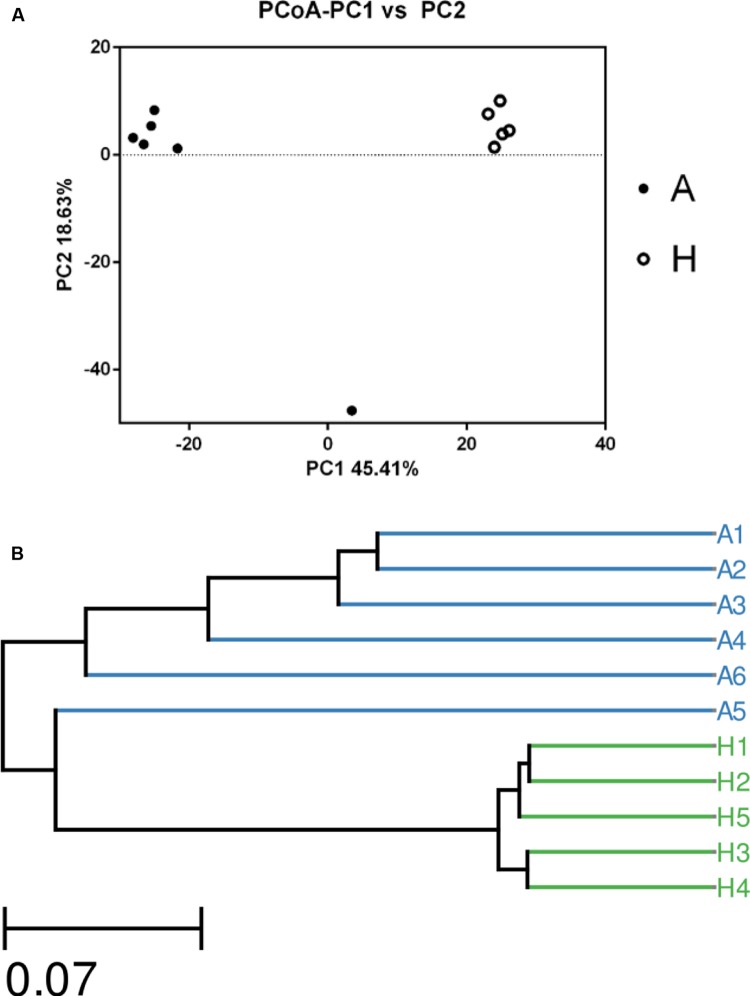
**The Principle component analysis (PCA) (A) and UPGMA Method of Beta diversity index (B) of groups A and H**.

The overall picture of the microbial composition of the samples in group A and H was obtained by PCoA, based on the relative abundance profiles of bacterial taxa. As shown in **Figure 2**, 5/5 samples in group H clustered together on the right upper of the coordinate axis, and 5/6 samples in group A gathered together on the left upper of the coordinate axis, and samples in group H were obviously deviated from the samples in group A (**Figure 2A**), which was future confirmed by UPGMA method (**Figure 2B**).

### Composition of the Bacterial Communities at Genus Level

**FIGURE 3:**
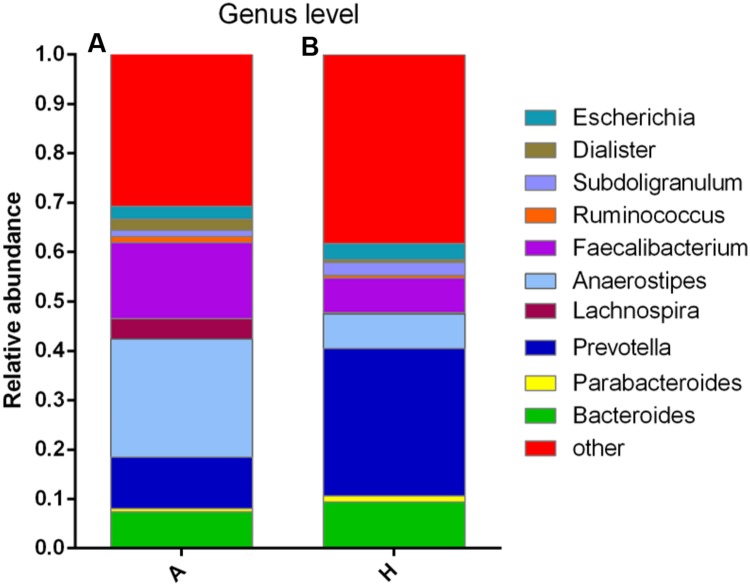
**Composition and relative abundance of bacterial communities based 16S rDNA sequences in A and H groups. (A)** Unsupervised hierarchical clustering analysis. **(B)** The relative abundances of the major bacteria in genus level.

At the genus level, data of top 10 microorganism populations was analyzed. As shown in **Figure 3**, *Bacteroides*, *Faecalibacterium*, *Anaerostipes*, *Prevotella*, and *Escherichia* constituted five common dominant genus in group A and H (7.38 vs. 9.41%, 15.32 vs. 7.02%, 23.9 vs. 7.0%, 10.42 vs. 29.86%, 2.57 vs. 3.24%), which accounted for 59.59 and 56.53% of the total sequencing number, and the bacteria did not belong to the dominant bacteria in these two groups and classified as the “others” had occupied 30.77 and 38.29%. In addition, the average ratios of *Bacteroides*, *Faecalibacterium*, *Anaerostipes*, *Prevotella*, *Escherichia*, and *Lachnospira* between groups A and H were 0.78, 2.18, 3.41, 0.35, 0.79, and 13.07 (**Figure 3**).

### Relative Abundance of the Bacterial Communities in Each Sample

**FIGURE 4:**
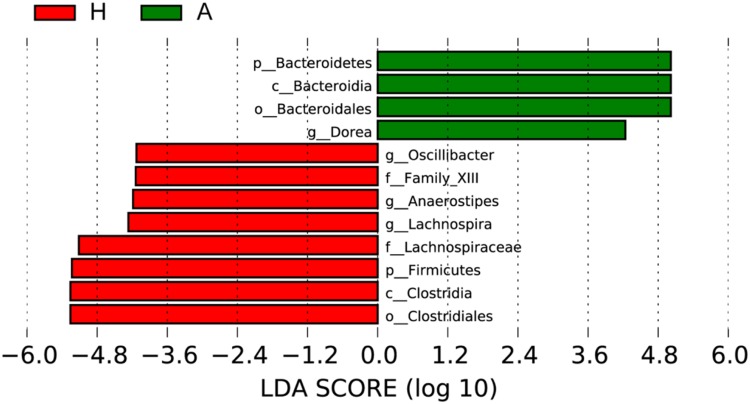
**Supervised comparison identifies differential abundance of bacteria using LEfSe (LDA > 4.0)**.

To determine the significant increased bacteria in group A or H, supervised comparisons by LEfSE (LDA > 4.0) were performed. In **Figure 4**, *Lachnospiraceae* (at family level), *Firmicutes* (at phylum level), *Clostridia* (at class level), *Oscillibacter* (at genus level), *Family XIII* (at family level), *Anaerostipes* (at genus level), *Lachnospiraceae* (at genus level) and *Clostridiales* (at order level) in group H were significant higher than that in group A, while *Bacteroidetes* (at phylum level), *Bacteroidia* (at class level), *Bacteroidales* (at order level), *Dorea* (at genus level) were significant higher than that in group H.

## Discussion

Accumulating clinical- and scientific research-based evidence is driving our increased awareness of the significance of the human microbiome (HM) to the healthy and homeostatic operation of the human CNS (Grenham et al., 2011; Hill et al., 2014; Fang, 2015; Kennedy et al., 2016). ALS belongs to neurodegenerative disease characterized by the loss of motor neurons (Scarrott et al., 2015), and the prevalence rate for ALS is substantially lower at 3.9/100000 in the United States (Mehta, 2015). To date, the pathogenesis of ALS remains unclear and is likely multifactorial, and the pathophysiology of ALS may be related to the gastrointestinal tract. The gut microbiota, which is also referred to as the second brain, may affect brain activity through the gut-microbiota–brain axis under both physiological and pathological conditions (Mayer et al., 2015; Sampson and Mazmanian, 2015), and accumulating evidence suggests that microbiota changes in the gastrointestinal tract of individuals possessed strong connection with neurological diseases and specifically, neurodegenerative diseases (Catanzaro et al., 2015).

In this study, high-throughput sequencing technology was used to compare the microbiota in intestinal tract of healthy people and ALS patients. To evaluate the tag quality, the raw tags, clean tags, average bp and OTUs in per sample were compared, and the mean number of 72972.36 clean tags, average length of 453 bp (**Table 1**), and the saturated Chao1 index and rarefaction curve ensured their reliability for the future analysis (Supplementary Figure S1).

In **Figure 1**, the Venn figure reflected a high percent of 43.78% of common OTUs in H group, and a low percent of 17.56% of common OTUs in A group, which indicated that the ALS, together with individual physiological status, had severely changed the microbial composition in patients feces, which deviating from the normal microbiota and characterized by the overgrowth of total OTU number and low percent of common OTUs. Moreover, the clustered samples A1, A2, A3, A4, and A6, as well as the clustered samples H1, H2, H3, H4, and H5 using PCoA and UPGMA method further conformed the microbial changes in feces of ALS patients (**Figure 2**).

Then, the top 10 microorganism populations were analyzed at genus level, and average ratios of *Bacteroides*, *Faecalibacterium*, *Anaerostipes*, *Prevotella*, *Escherichia*, and *Lachnospira* between groups A and H were 0.78, 2.18, 3.41, 0.35, 0.79, and 13.07 (**Figure 3**). Furthermore, supervised comparisons by LEfSE (LDA > 4.0) were performed to find the significant changed bacteria, and the relative richness of *Firmicutes* at phylum level, *Clostridia* at class level, *Clostridiales* at order level, *Lachnospiraceae* and *Family XIII* at family level, *Oscillibacter*, *Anaerostipes* and *Lachnospiraceae* at genus level in group H were significant higher than that in group A, while *Bacteroidetes* at phylum level, *Bacteroidia* at class level, *Bacteroidales* at order level and *Dorea* at genus level were significant higher in group A (**Figure 4**).

In healthy adults, 80% of the identified fecal microbes can be classified into three dominant phyla: *Bacteroidetes, Firmicutes*, and *Actinobacteria*, and the *Firmicutes* to *Bacteroidetes* ratio is regarded to be of significant relevance with human health (Mariat et al., 2009), and the significant increase of *Firmicutes* in H group and significant increase of *Bacteroidetes* in A group indicated that the ALS has seriously influenced patients’ healthy, characterized by the decreased *Firmicutes/Bacteroidetes* ratio. At genus level, the *Dorea* in group A was significant higher than that in healthy people, whose major end products of glucose metabolism are ethanol (Vos et al., 2011). Moreover, the significant decrease of *Oscillibacter* (was found in significantly more samples from healthy control test subjects than from patients diagnosed with Crohn’s disease; Mondot et al., 2011), *Anaerostipes* (represents more than 2% of total colonic microbiota in the healthy colon, and are believed to play an important functional role in the gut ecosystem due to their ability to produce butyrate from lactate; Bui et al., 2014) and *Lachnospiraceae* (can protect from colon cancer in humans by producing butyric acid; Meehan and Beiko, 2014) in group A further confirmed the interaction of ALS with intestinal microbiota.

In summary, we found that host microbiota were markedly different in health and disease, and the overgrowing of pathogens and reduction of probiotic organisms in intestines of ALS patients might up-regulated or down-regulated the production of NO, GABA, SCFAs, and LPS, which eventually increased the pathogenesis of ALS, and the ALS conversely aggravated the imbalances of intestinal microbiota, causing a vicious circle for host health. In the present study, we provide basic data to clarify the key bacteria during disease occurring, which may assist our understanding and treatment of ALS by inhibiting the growth of pathogens and enhancing the number of probiotics.

## Author Contributions

TC designed the experiment; XF, XnW, SY, FM, and XaW performed the experiments; TC and HW analyzed the data and wrote the manuscript. All authors discussed the results and commented on the manuscript.

## Conflict of Interest Statement

The authors declare that the research was conducted in the absence of any commercial or financial relationships that could be construed as a potential conflict of interest.

## Footnotes

1 http://metastats.cbcb.umd.edu/
