# Supplementary material for: Evaluation of the Microbial Diversity in Amyotrophic Lateral Sclerosis Using High-Throughput Sequencing
Source: Front Microbiol. 2016 Sep 20;7:1479. doi: 10.3389/fmicb.2016.01479 (PMC5028383; doi:10.3389/fmicb.2016.01479)
Supplement: Supplementary file 1 [file Data_Sheet_1.DOCX]

[Supplementary materials](http://dict.youdao.com/w/supplementary%20materials/#keyfrom=E2Ctranslation) 1

Table 1 The information of health people and patients

| NO. | age | sex | antibiotics |
| --- | --- | --- | --- |
| A1 | 63 | male | NO |
| A2 | 49 | male | NO |
| A3 | 52 | male | NO |
| A4 | 77 | male | NO |
| A5 | 52 | female | NO |
| A6 | 48 | male | NO |
| H1 | 45 | male | NO |
| H2 | 55 | male | NO |
| H3 | 52 | female | NO |
| H4 | 51 | female | NO |
| H5 | 45 | female | NO |

[Supplementary materials](http://dict.youdao.com/w/supplementary%20materials/#keyfrom=E2Ctranslation) 2


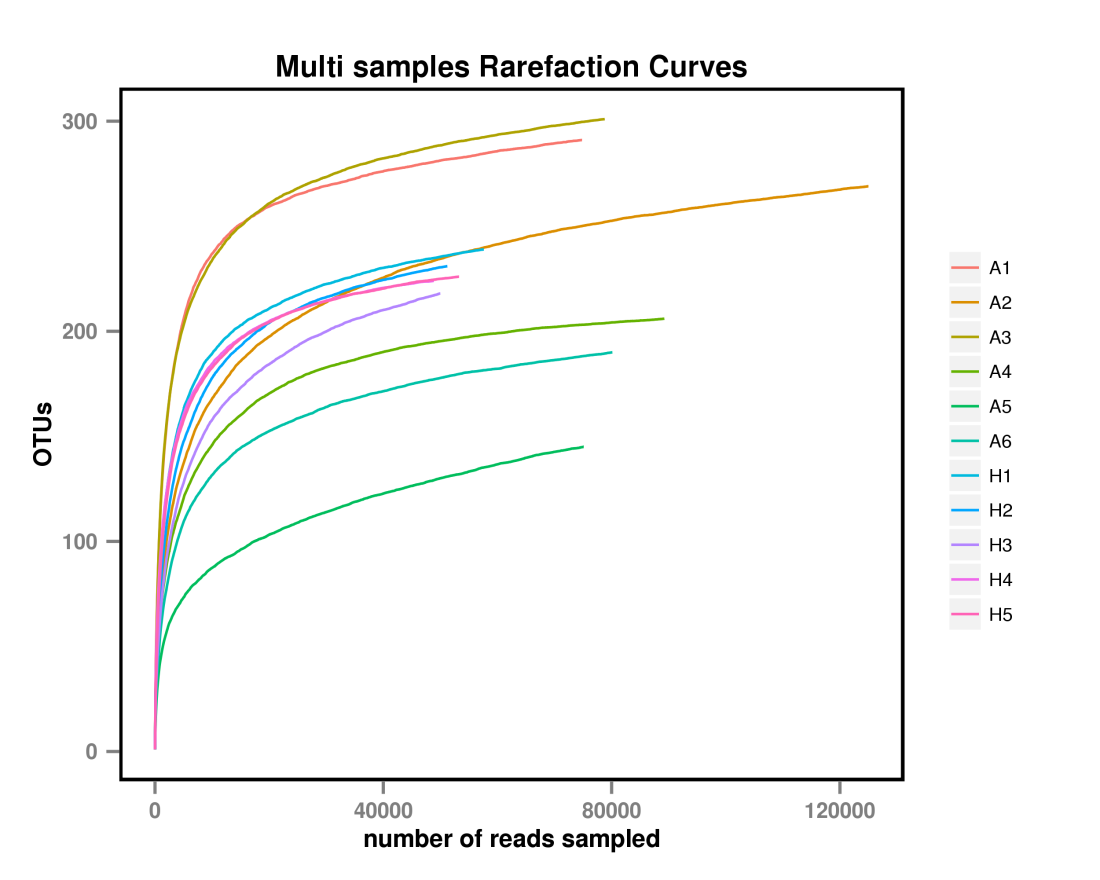

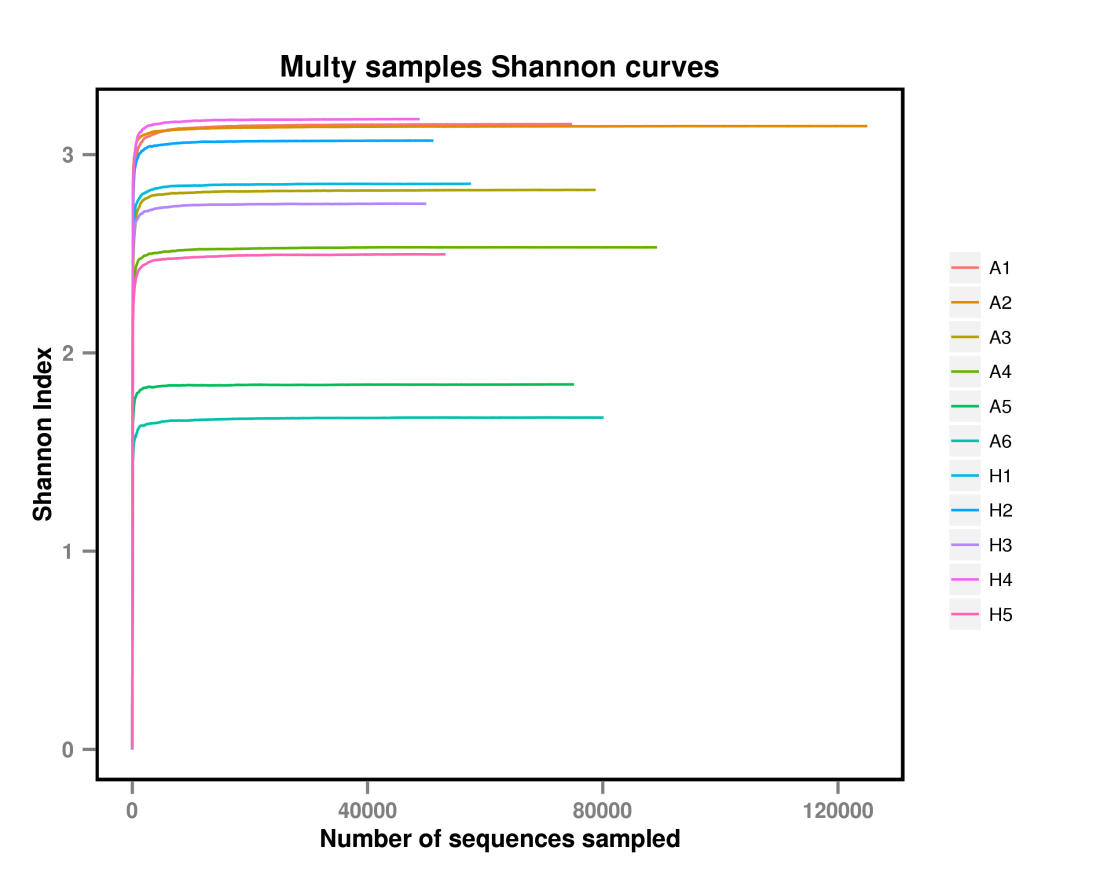


Fig 1 The rarefaction curves and Shannon curves of groups A and H
